# Certified Fellowship in Abdominal Wall Surgery—A Collaboration Between the UEMS and the European Hernia Society

**DOI:** 10.3389/jaws.2024.12945

**Published:** 2024-04-22

**Authors:** Ferdinand Köckerling, Salvador Morales-Conde, Maarten Simons, Daniel Casanova, Barbora East, Andrew de Beaux, Nadia Henriksen, Sebastian Roka, Arthur Felice

**Affiliations:** ^1^ Hernia Center, Vivantes Humboldt Hospital, Academic Teaching Hospital of Charité University Medicine, Berlin, Germany; ^2^ Department of Surgery, University Hospital Virgen Macarena Seville, Seville, Spain; ^3^ OLVG Hospital, Amsterdam, Netherlands; ^4^ Department of Surgery, University of Cantabria, Santander, Spain; ^5^ 3rd Department of Surgery and 1st Medical Faculty, Charles University at Motol University Hospital, Prague, Czechia; ^6^ NHS Lothian and Oxford University Hospitals, University of Edinburgh Sire Murrayfield Hospital, Edinburgh, United Kingdom; ^7^ Department of Hepatic and Gastrointestinal Disease, Herlev Hospital, University of Copenhagen, Herlev, Denmark; ^8^ Wiener Gesundheitsverbund, Klinik Donaustadt, Vienna, Austria; ^9^ Faculty of Medicine and Surgery, University of Malta, Msida, Malta

**Keywords:** abdominal wall surgery, fellowship, examination, hernia specialists, education

## Abstract

**Background:** Abdominal wall surgery (AWS) is characterised by the increasing caseload and the complexity of the surgical procedures. The introduction of a tailored approach to AWS utilising laparoendoscopic, robotic and/or open techniques requires the surgeon to master several surgical techniques. All of which have an associated learning curve, and the necessary knowledge/experience to know which operation is the right one for the individual patient. However, the reality in general surgery training shows that training in just a limited number of procedures is not enough. By the end of general surgery training, many chief residents do not feel they are yet ready to carry out surgery independently. Therefore, hernia surgery experts and societies have called for the introduction of a Fellowship in Abdominal Wall Surgery.

**Methods:** The UEMS (Union Européenne des Médecins Spécialistes, European Union of Medical Specialists) in collaboration with the European Hernia Society (EHS) introduced a fellowship by examination in 2019. As a prerequisite, candidates must complete further training of at least 2 years with a special focus on abdominal wall surgery after having completed their training in general surgery. To be eligible for the examination, candidates must provide evidence of having performed 300 hernia procedures. In addition, candidates must have accrued sufficient “knowledge points” by attending abdominal wall surgery congresses, courses and clinical visitations, and engaged in scientific activities. On meeting the requirements, a candidate may be admitted to the written and oral examination.

**Results:** To date, three examinations have been held on the occasion of the Annual Congress of the European Hernia Society in Copenhagen (2021), Manchester (2022) and Barcelona (2023). Having met the requirements, 48 surgeons passed the written and oral examination and were awarded the *Fellow European Board of Surgery*—*Abdominal Wall Surgery* certificate. During this time period, a further 25 surgeons applied to sit the examination but did not fulfil all the criteria to be eligible for the examination. Fifty experienced abdominal wall surgeons applied to become an Honorary *Fellow European Board of Surgery*—*Abdominal Wall Surgery*. Fourty eight were successful in their application.

**Conclusion:** The Fellowship of the European Board of Surgery - Abdominal Wall Surgery by examination has been successfully introduced at European level by the joint work of the UEMS and the EHS. The examination is also open to surgeons who work outside the European area, if they can fulfil the eligibility criteria.

## Introduction

With more than 20 million operations every year, abdominal wall hernia repair is one of the most common procedures in general surgery [1]. In particular, over 1.1 million abdominal wall hernia repairs are performed in the United States, 375,000 in Germany and 100,000 in the United Kingdom every year [2–4]. The number of patients with abdominal wall hernias is expected to rise in the future for a number of reasons, including the effects of an aging population and the obesity epidemic [2]. The complexity of abdominal wall hernias and thus the challenges of repair, has risen dramatically in recent decades [5].

The introduction of a tailored approach in laparoendoscopic, robotic and new open techniques requires the surgeon to master several surgical techniques, all of which have a relevant learning curve [6]. Besides, today increasingly more patients with risk factors and diagnoses likely to impact outcomes are undergoing surgery [6]. Around one-third of all patients with inguinal or incisional hernia have several risk factors for an unfavorable outcome [6]. As such, there are many reasons for the increasing complexity of abdominal wall surgery: the introduction of new techniques, more difficult cases and a recognised tailored approach. In addition, there is increasing public awareness that demands nothing short of optimal treatment results [6]. Doing the right operation at the right time on the right patient and doing it right is important to every branch of surgery.

The reality of general surgery is in sharp contrast with the more stringent demands made on abdominal wall surgery. While trainee operation volume varies by hospital and country, training in general surgery typically comprises 50–100 hernia repairs, of which only around 25 are laparoendoscopic procedures [6]. There is increasing evidence that in abdominal wall surgery, there is a relationship between a surgeon’s caseload and the patient outcome [7–9]. By the end of general surgery training, many chief residents do not feel they are yet ready to carry out surgery independently [10–14], highlighting the need for additional training and specialisation [13]. Over 80% of chief residents undergo a 1 to 2 year fellowship in various subspecialties [13]. In light of the trends described above, scientific associations and experts have for years been calling for the introduction of fellowships in abdominal wall surgery [5, 15–22].

The UEMS was the first medical institution in the world to introduce certification of a fellowship in abdominal wall surgery in 2021, by setting minimum standards of operative experience, academic activities in relation to AWS, and the successful passing of the UEMS AWS examination. This paper reports on the requirements to be met to be awarded *Fellowship in Abdominal Wall Surgery* as well as the initial experiences in the certification process.

## UEMS (Union Européenne des Médecins Spécialistes, European Union of Medical Specialists)

The UEMS is an international non-profit organization under Belgian law [23]. The UEMS was founded in 1958. It is the oldest and largest international medical institution in Europe and today represents the interests of some 1.6 million medical specialists. It is supported by 41 National Medical Associations, representing the interests of medical specialists in their respective countries (Table 1). There are also collaborations with other National Medical Associations (Table 2).

**TABLE 1:** UEMS—Full National Member Associations.

**➢ **Austria	Austrian Medical Association
**➢ **Belgium	Groupement des Unions Professionelles Belges de Médecins Spécialistes
**➢ **Bulgaria	Bulgarian Medical Association
**➢ **Croatia	Croatian Medical Association
**➢ **Cyprus	Cyprus Medical Association
**➢ **Czech Republic	Czech Medical Association
**➢ **Denmark	Danish Medical Association
**➢ **Estonia	Estonian Medical Association
**➢ **Finland	Finnish Medical Association
**➢ **France	Avenir Spé
**➢ **Germany	Spitzenverband Fachärzte Deutschland
**➢ **Greece	Panhellenic Medical Association
**➢ **Hungary	Association of Hungarian Medical Societies
**➢ **Iceland	Icelandic Medical Association
**➢ **Ireland	The Irish Medical Organisation
**➢ **Italy	Federazione Nazionale degli Ordini dei Medici
**➢ **Latvia	Latvian Medical Association
**➢ **Lithuaria	Lithuanian Medical Association
**➢ **Luxembourg	Association des Médecins et Médecins - Dentistes
**➢ **Malta	The Medical Association of Malta
**➢ **Netherland	Federatie Medisch Specialisten
**➢ **Norway	Norwegian Medical Association
**➢ **Poland	Polish Chamber of Physicians and Dentists
**➢ **Portugal	Portuguese Medical Association
**➢ **Romania	Romanian Medical Association
**➢ **Slovakia	Slovak Medical Association
**➢ **Slovenia	Medical Chamber of Slovenia
**➢ **Spain	General Medical Council of Spain
**➢ **Sweden	Swedish Medical Association
**➢ **Switzerland	Swiss Medical Association
**➢ **United Kingdom	British Medical Association

**TABLE 2:** UEMS—Associate Member and Observer National Association.

**➢ **Armenia	Armenian Medical Association
**➢ **Israel	Israel Medical Association
**➢ **Serbia	Serbian Medical Association
**➢ **Turkey	Turkish Medical Association
**➢ **Ukraine	Ukrainian Medical Society
**➢ **Georgia	Georgian Association of Medical Specialists
**➢ **Iraq	The Arab Board for Health Specialisations in Iraq
**➢ **Lebanon	Lebanese Order of Physicians
**➢ **Morocco	Collége Syndical National Des Médecins Spécialistes Prives
**➢ **Tunisia	Ordre des Medecines de Tunisie

The principle goals of the UEMS are:

• Representation of all medical specialists in Europe
• Harmonisation of training for medical specialists in Europe
• Development of standards for all medical disciplines

The 41 National Medical Associations nominate the delegates and heads of delegates for the UEMS Bodies (Executive Committee, Council, Specialist Sections, Divisions/Working Groups). Reflecting the growing trend towards specialisation, the UEMS now includes 43 medical disciplines, 15 multidisciplinary committees and over 20 divisions and working groups.

The individual medical disciplines, Multidisciplinary Joint Committee, Divisions and Working Groups have formed a subgroup with the European Board, which together with the respective European scientific association are responsible for education and training.

The entire field of surgery is represented in the UEMS by the following sections: cardiovascular and thoracic surgery, neurosurgery, oral maxillofacial surgery, orthopedics and trauma surgery, pediatric surgery, plastic, reconstructive and esthetic surgery, thoracic surgery, vascular surgery and general surgery.

Topics related to several disciplines are addressed by the UEMS in the Multidisciplinary Joint Committees (hand surgery, intensive care medicine, oncology, phlebology, spinal surgery, sports medicine, wound management, oesophageal surgery).

Within the UEMS Section of Surgery, the subspecialties are represented by the Divisions/Working Groups [24]. In collaboration with the respective European Board of Surgery and the appropriate European scientific associations, they define the minimum requirements for the completion of surgical training. On the successful completion of training (in both general surgery and the subspeciality) as laid out in the UEMS requirements, the candidate then takes a written and oral examination. If the candidate passes the examination they are awarded a certificate documenting they have adequate knowledge and proven expertise in the subspecialty area. The certificate then bears the title *Fellow European Board of Surgery—Subspecialty*.

Through its Divisions and Working Groups, the UEMS Section of Surgery has many years of experience in organising training fellowships and subsequent examinations in general surgery and its subspecialties (general surgery, breast surgery, surgical oncology, endocrine surgery, coloproctology, transplantations, hepatopancreatobiliary surgery, minimally invasive surgery, trauma surgery, emergency surgery, etc.).

## Fellowship in Abdominal Wall Surgery

In 2019, the UEMS Section of Surgery together with the European Board of Surgery introduced the qualification Fellow European Board of Surgery—Abdominal Wall Surgery (FEBS-AWS) A cooperation agreement was concluded with the UEMS and the EHS to implement this [25]. The requirements for clinical training, theoretical knowledge and the examination content were then jointly defined. Candidates must have completed at least 6 years of training in general surgery [26] (number of years to the completion of General Surgery training will vary by country). Once completed General Surgery training, candidates should undertake at least two-years of further training/experience with a special focus on AWS.

To meet the requirements governing the European Board of Surgery qualification for abdominal wall surgery, training should take place under the instruction of more than one principle trainer and one hospital or institution. Two trainers must declare that to their knowledge the information provided by the candidate concerning their training experience in AWS is correct [27].

The AWS syllabus comprehensively describes the knowledge and skills mandatory for the qualification FEBS AWS [28] (Table 3a–c).

**TABLE 3:** (A) Abdominal Wall Surgeon Knowledge (I). (B) Abdominal Wall Surgeon Knowledge (II). (C) Abdominal Wall Surgeon Knowledge (III).

The specialty of Abdominal Wall Surgery requires documented and assessed knowledge in:
Basic knowledge of abdominal wall
• Basic anatomy of the groin
• Basic anatomy of the abdominal wall
• Physiopathology of the abdominal wall
Prevention of abdominal wall hernias
• Prevention of incisional and parastomal hernias following open abdominal procedures by small bite suture technique and/or prophylactic mesh
• Risk factors (smoking, obesity, pulmonary disease)
Diagnostic
• Differentiated pre- and postoperative use of diagnostic procedures (clinical examination, CT, MRI, ultrasound)
Indication for abdominal wall surgery and choice of technique
• Indication for abdominal wall surgery under consideration of the current guidelines
• Tailored approach in elective and emergency abdominal wall surgery under consideration of gender, bilaterality, recurrence and other factors
Preoperative management
• Physical examination
• Exact information on previous operations including mesh or other implants
• Tests of respiratory, cardiac, renal and endocrine function
• Patient information and documentation of informed consent (including risks of technique)
• Information about principles of ERAS
• Prophylaxis of thromboembolic disease
• Antibiotic prophylaxis
• Assessment of fitness for anaesthesia and surgery
• Premedication and sedation
• Nutritional evaluation of the patient
• Preoperative conditioning of patient with risk factors
Intraoperative care
• Patient positioning (including extreme anti-Trendelenburg and other positions)
• Prevention of nerve and other injuries in the anesthetized patient
• Principles of general and regional anaesthesia (including optimal fluid management)
• Prevention of medical and surgical complications

Postoperative management
• Pain control
• Post-operative monitoring
• Post-operative complications
• Prevention, recognition and management of complications
• Early recognition and management of sepsis
• Respiratory failure-recognition and treatment
• Nutritional support-indications, techniques, total parenteral nutrition
• ERAS protocol
• Progressive return to activity
Basic abdominal wall surgical technique and technology
• Patients’ positioning
• Surgical instruments and technical OR equipment for open or laparoendoscopic access
• Instruments and technical equipment for AWS (especially cameras, light sources, insufflators, energy devices)
• Techniques of establishing access for AWS (open and laparoendoscopic)
• Detection and treatment of intraoperative complications
• Trocar positions, placement and closure techniques
• Basic ergonomics in AWS (table and monitor position)
• Suturing, stapling and sealing in AWS
• Surgical meshes
• Diathermy-principles and precautions and principles of energy sources
• Explosion hazards relating to general anaesthesia and endoscopic surgery
• Role of robotic in AWS
Abdominal wall anatomy
The surgical anatomy of the abdominal wall, abdominal cavity and its viscera and applied physiology of the alimentary system, relevant to clinical examination, interpretation of special investigations, understanding of disorders functional and treatment of abdominal wall disease.
Abdominal wall hernia types
• Inguinal hernia
• Femoral hernia
• Primary ventral hernia (umbilical, epigastric)
• Rectus diastasis
• Incisional hernia
• Miscellaneous hernias
• Parastomal hernia
Procedures inguinal hernia
• Laparoscopic vs open repair
• Lichtenstein mesh repair
• Open preperitoneal hernia repair
• Shouldice non-mesh repair
• Total preperitoneal repair (TEP)
• Transabdominal preperitoneal repair (TAPP)
• Inguinal hernia repair in the context of other surgical procedures
• Inguinal hernias in emergency

Procedures ventral hernia
• Laparoscopic vs open repair
• Open sublay
• Open onlay
• Other open alternative (intraperitoneal, sandwich techniques)
• Laparoscopic intraperitoneal onlay mesh (IPOM)
• New Minimally Invasive Techniques and approaches: MILOS, e-TEP, LIRA, and ELAR
• Open anterior and posterior component separation technique
• Laparo-endoscopic anterior and posterior component separation
• Option for parastomal hernia repair
• Option for lateral hernias and those close to bone margins
• Ventral hernia repair during bariatric surgery or other concomitant procedures
• Ventral hernias in emergency
Evaluation & Quality Control
• Decision-making in surgery
• Clinical audit
• Statistics and computing in surgery
• Documentation
• Principles of research and design and analysis of clinical trials
• Principles of experimental research in AWS
• Critical evaluation of innovations-technical and pharmaceutical
• Principles and pharmacology of intravenous drug delivery
• Quality control and quality management
• CIRS (Critical Incident Reporting System)
• Implementation of clinical studies
• Legal aspects
• Communication with patients, relatives and colleagues
• Team working, leadership
• Situational awareness, stress and fatigue

This syllabus should not be viewed as static but will be continuously revised and up-dated by the members of the Executive Committee as necessary. The candidates are expected to update their knowledge and skills level according to the recent surgical practice and scientific literature.

## Eligibility Requirements for the Examination

Apart from the training periods, training institutions and trainers, minimum requirements are defined for the caseloads, participation in congresses, courses, clinical visitations as well as in scientific activities in the field of AWS [27].

Minimum requirements are defined for the caseloads (Category A = inguinal hernias, B = primary ventral hernias, C = incisional hernias) and participation in congresses, courses, clinical visitations as well as scientific activities in the field of abdominal wall surgery (D) [27]. For the FEBS-AWS qualification the candidate needs 800 credit points in the various categories (Tables 4, 5) [27].

**TABLE 4:** EBSQ AWS catalogue procedures and options.

Category A: Inguinal hernia repairs^a^	*n* = 200
1. Primary inguinal hernia repair in TAPP, TEP, Lichtenstein, open preperitoneal repair or Shouldice technique	*n* = 125
2. Bilateral inguinal hernia repairs	*n* = 20
3. Female groin hernia repair	*n* = 20
4. Recurrent inguinal hernia repair	*n* = 20
5. Scrotal hernia repair	*n* = 5
6. Emergency inguinal hernia repair	*n* = 5
7. Inguinal hernia repair following previous lower abdominal and pelvic surgery	*n* = 5

Category B: Primary ventral hernia ± rectus diastasis repairs^b^	*n* = 50
1. Open suture, open mesh or laparoendoscopic umbilical hernia repair	*n* = 30
2. Open suture, open mesh or laparoendoscopic epigastric hernia repair	*n* = 10
3. Open mesh or laparoendoscopic umbilical and epigastric hernia plus rectus diastasis repair	*n* = 5
4. Emergency umbilical and epigastric hernia repair	*n* = 5

Category C: Incisional and complex hernia repairs^c^	*n* = 50
1. Incisional hernia repair in laparoscopic IPOM, open sublay, open onlay or component separation technique	*n* = 40
2. Open mesh or laparoendoscopic recurrent incisional hernia repair	*n* = 5
3. Emergency incisional hernia repair	*n* = 3
4. Parastomal hernia repair	*n* = 2

^a^ The 50% rule: at least 50% of a total number of 500 credit points (c.p.) have to be achieved as principle surgeon (min 100 procedures).The total number of 400 credit points for Category A is mandatory.

^b^ The 75% rule: At least 75% of a total number of 100 c.p. have to be achieved as principle surgeon (minimum 38 procedures as principle surgeon = 76 c.p.)

^c^ The 100% rule: All procedures in this category have to be performed as principle surgeon (50 procedures = 100 c.p.)

**TABLE 5:** Category D—200 points for training, education, clinical visits and research.

200 points for training, education and research in abdominal wall surgery Activity	Credit points
Participation in national AWS congresses	4
Poster presentation (first author) at national AWS congresses	6
Poster presentation (co-author) at national AWS congresses	3
Oral presentation (presenting author) at national AWS congresses	8
Oral presentation (co-authors) at national AWS congresses	5
Participation at recognized international AWS congresses	8
Poster presentation (first author) at recognised international AWS congresses	16
Poster presentation (co-author) at recognised international AWS congresses	8
Oral presentation (first author) at recognised international AWS congresses	20
Oral presentation (co-author) at recognised international AWS congresses	10
AWS Publication (first/corresponding author) in peer reviewed national surgical journals	20
AWS Publication (co-author ) in peer reviewed national surgical journals	10
AWS Publication ( first/corresponding author ) in peer reviewed international surgical journals	40
AWS Publication (co-author ) in peer reviewed international surgical journals	20
Participation in a recognized AWS postgraduate course	12
Participation in a hands-on AWS course under the leadership of a recognized expert in abdominal wall surgery	12
Clinical visits or participation in fellowship-programs at recognized abdominal wall surgery centres	5 per day

For the principle surgeon 2 credit points are given for each operation in categories A, B, and C [27]. As 600 credit points are needed in Categories A, B and C, 300 procedures must be performed by the candidate as principle surgeon [27]. In categories A and B one credit point can be gained as first assistant of a recognised expert, but is limited to 100 procedures in category A and 12 operations in category B [27]. In this case, 112 additional procedures as assistant surgeon needs to be performed [27]. All procedures of category C must be done as principle surgeon [27].

Candidates must demonstrate skills in each of the described areas of responsibility and be able to present a complete and signed log book. In the log book the patient’s initials or hospital admission number, type of procedure, date of procedure and approval with signature by independent experts have to be provided for each item. The individual log books are scrutinised in the eligibility process. In addition, the candidate must demonstrate that they have engaged in scientific activities related to abdominal wall surgery and participated in congresses or courses and/or work shadowing in accredited hernia centers. (Table 5) The eligibility criteria also include submission of curriculum vitae.

## Examination

Only those who meet the above requirements may be admitted to the examination [27]. Applications are reviewed by the Executive Committee of the Abdominal Wall Surgery Working Group. Applications must be submitted for assessment on the internet platform^1^. To date, examinations have been held on the day prior to the start of the Annual Congress of the EHS, in the English language. (In future, the UEMS plans to hold examinations in other languages.) The examination comprises a written and an oral part. In the written part, candidates must answer 100 multiple choice questions within 4 h.

The purpose of the objective structured clinical examination (OSCE) circuit is to evaluate process thinking and judgement and the focus is on decision-making. In the OSCE circuit candidates should be able to answer what they would do and how and why. The circuit consists of at least 6 stations (10 min each, with total duration of the circuit: 60 min). All examiners are surgeons in active practice and hold the certificate FEBS-AWS. A total of 600 points can be achieved in the Board Examination, 300 points in the MCQ test (3 points per question) and 300 points in the OSCE circuit (50 points per station). The threshold for passing the examination is 75%, which means at least a total of 450 points. Candidates are expected to have a comprehensive knowledge of the entire field of AWS as this basis for the examination.

## Certificate

**FIGURE 1:**
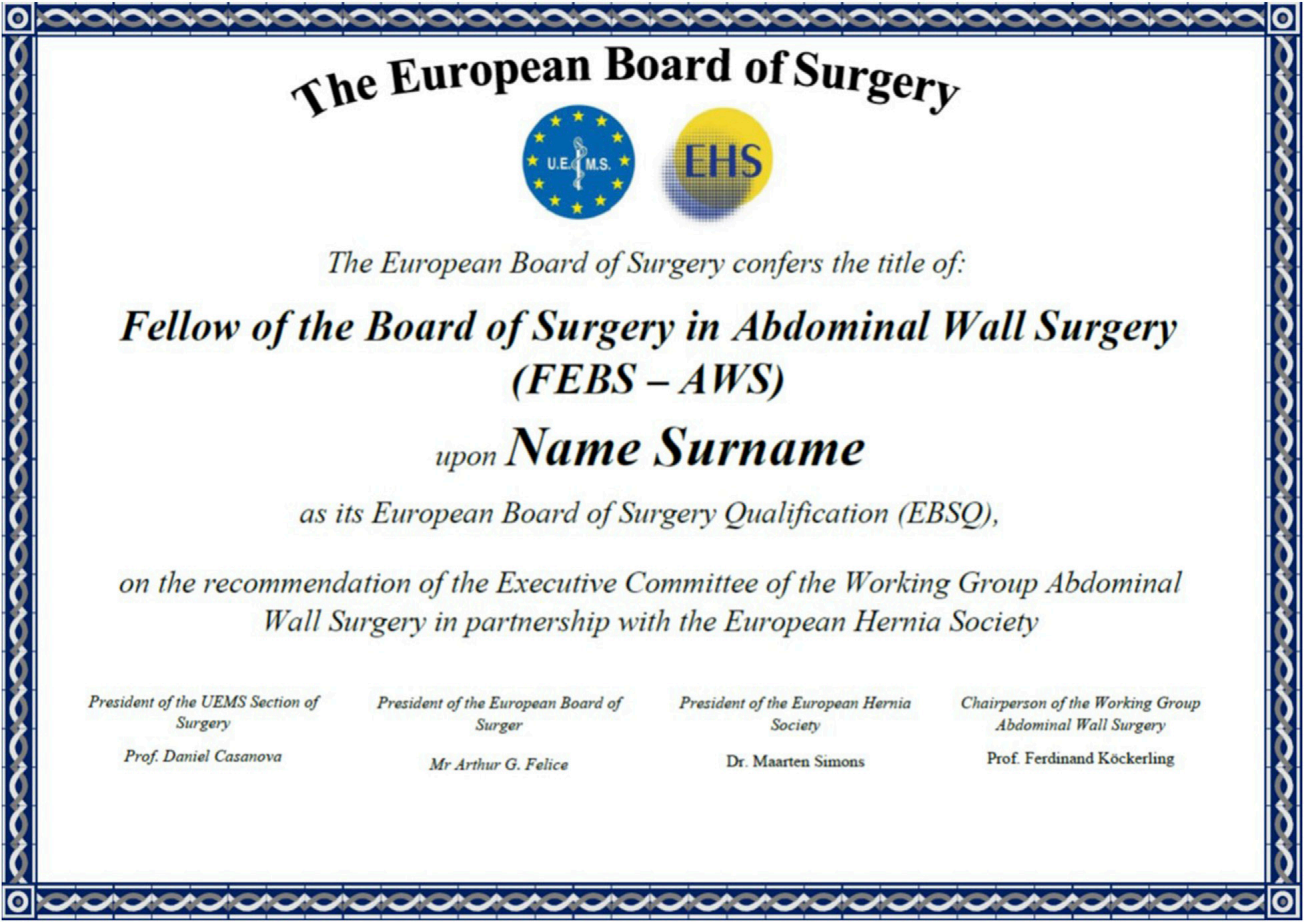
Certificate for the title “Fellow of the Board of Surgery in Abdominal Wall Surgery” (FEBS-AWS).

The results of the board examination are announced within 1 h of the end of the last circuit. Successful candidates are awarded their FEBS AWS certificates during the Annual Congress of the EHS. The candidate is awarded the title *Fellow European Board of Surgery in Abdominal Wall Surgery (FEBS AWS).* The certificate is signed by the president of the UEMS Section of Surgery, president of the European Board of Surgery, president of the European Hernia Society and the chairperson of the Abdominal Wall Surgery Working Group (Figure 1).

## Previous and Future Examinations

The first examination for the FEBS-AWS qualification was held in Copenhagen on 12 October 2021. Thirteen of 21 candidates (failure rate: 38.1%) met the eligibility criteria and were admitted to the examination. All 13 candidates passed the examination, gaining at least 75% of the total credit points of the maximum 600 points that can be awarded.

The second examination was held in Manchester on 17 October 2022. Twenty-two of 32 candidates (failure rate: 31.1%) met the requirements for admission to the examination. All 22 candidates passed the examination.

The third examination was held in Sitges, Barcelona on 2 and 3 May 2023. Thirteen of 20 candidates (failure rate: 35.0%) met the criteria for admission to the examination. All 13 candidates passed the examination.

The main obstacle to admission to the written and oral examination were the, required surgical caseload and in particular, failing to amass the required credits in Section D, namely participation in hernia congresses, scientific activities, courses and short clinical attachments. Candidates who passed the eligibility assessment appear to have sufficient knowledge and skills to pass the examination. Therefore, all those interested in obtaining the FEBS AWS certificate are called upon to carefully check whether all requirements are met before submitting their application.

The 48 successful candidates awarded the title FEBS AWS so far are from the following countries: Germany *n* = 7, UK *n* = 6, Spain *n* = 5, Austria *n* = 4, Portugal *n* = 4, Switzerland *n* = 4, Belgium *n* = 3, Denmark *n* = 3, Netherlands *n* = 2, Sweden *n* = 2, Italy *n* = 2, Romania *n* = 2, Greece *n* = 1, Czech Republic *n* = 1, Turkey *n* = 1 and Poland *n* = 1.

The next examination will be held on 28 May 2024 in Prague, the day before the International Congress of the European Hernia Society from 29–31 May 2024. There are also plans to hold the examination for the first time in German on 6 November 2024 in Leipzig. A survey of the 1,200 members of the German Hernia Society revealed that over 70 candidates would like to take the examination but do not feel confident doing so in English. In the interest of the further development of AWS, more surgeons qualified in AWS are urgently needed. In the future, candidates working outside the 41 countries that make up UEMS can also aspire to achieving the title FEBS AWS, provided they meet the same requirements.

## Requirements for the Honorary Fellowship in Abdominal Wall Surgery

The Fellowship in AWS provided by the UEMS Section of Surgery, the European Board of Surgery, and the EHS is a new qualification [29]. To establish the FEBS AWS qualification in Europe as a standard, experienced surgeons in the field of AWS are needed as trainers and examiners. That was the rationale for the UEMS Section of Surgery and the European Board of Surgery deciding to introduce the FEBS AWS honorary certificate.

Surgeons with an interest in AWS are eligible to apply only if they have a minimum of 10 consecutive years of independent practice and/or experience in formally recognised hospitals, National Health Services or university posts.

The application must include:

• A cover letter highlighting the achievements of their career
• A complete updated Curriculum vitae
• A specific Curriculum vitae related to abdominal wall surgery
• Membership certification of an abdominal wall surgery association, such as EHS
• Letters from two peers of their choice who will explain in detail why they support the application

Each application is reviewed by the Executive Committee of the Abdominal Wall Surgery Working Group. If the criteria are met, the applicant will be awarded the FEBS AWS honorary certificate.

To date, the Executive Committee of the AWS Working Group has positively reviewed 48 applications for the FEBS-AWS honorary certificate. Surgeons awarded an honorary certificate are from following countries: Germany *n* = 15, Austria *n* = 5, UK *n* = 5, Netherlands *n* = 4, Switzerland *n* = 4, Poland *n* = 3, Denmark *n* = 2, Spain *n* = 2, Romania *n* = 2, Belgium *n* = 1, Turkey *n* = 1, Slovenia *n* = 1, Italy *n* = 1, Norway *n* = 1 and USA *n* = 1. The honorary fellows are expected to offer fellowships in abdominal wall surgery in their respective countries as well as actively participate in the development of the examination content and act as examiners. Two applications for honorary FEBS AWS were turned down, as the experience of the applications were similar to many who had undertaken the FEBS AWS examination.

## Discussion

“Over the last 20 years, a great number of innovations both in operative techniques and technologies have revolutionised abdominal wall surgery” [22]. Associated with this has been the increase in the complexity of ventral hernia repair [5, 15–22]. This has led to calls for AWS to become a recognised subspecialty in the United States and Europe [5, 15–22]. Despite this, certification around the attainment of a minimum standard in AWS have been offered neither in the USA nor in Europe to date [5, 30–32]. Therefore, the Fellowship in AWS (FEBS AWS) presented here is the first of its kind worldwide. The requirements for successful completion of the fellowship include at least 6 years of training in general surgery, at least 2 years of further clinical activity with a focus on abdominal wall surgery, independent performance of a minimum number of abdominal wall surgery procedures, a minimum number of credit points for scientific engagement in abdominal wall surgery activities, participation in congresses and courses and work shadowing as well as passing the written and oral examination. This training period is a minimum standard, and many of the surgeons who have taken the examination in the past three examination diets have spent more years in both training and post-training. It is acknowledged that surgical “training” never really ends.

The experience to date with the eligibility assessment for the FEBS AWS has shown that the requirements that candidates most often fail to meet are those in Section D of the examination application. Namely, engagement in scientific activities related to abdominal wall surgery, participation in congresses and courses as well as work shadowing experts in AWS. The surgeons who have meet all the eligibility requirements to date have all passed the FEBS AWS examination with relative ease, although for some, the academic viva is the one that has often had the lowest marks. The ability to critically appraise the medical literature remains an important skill for any practicing doctor.

We believe the introduction of a FEBS-AWS honorary certificate is an important requirement for the establishment of training “fellowships” in AWS, identifying centres for surgeons to visit, and the certification of surgeons who train in such centres. Only with the help and support of experienced abdominal wall surgeons, who are also active in the EHS, will it be possible to grow this new recognised subspecialty in general surgery. Another important role for those surgeons with an FEBS-AWS honorary certificate is the development of the written and oral examination questions in addition to acting as an examiner. It is no doubt difficult to confer on a candidate a title that one, as an examiner, does not possess oneself. While the scientific basis for the introduction of the Fellowships in Abdominal Wall Surgery by the UEMS Section of Surgery, European Board of Surgery and European Hernia Society was founded on the suggestions of the ACCESS Group [14], the need to continually update the syllabus in line with new insights, innovations and guidelines is evident. This applies to both the examination candidate and examiner, who must keep the required listed knowledge up to date by reading the literature, attending congresses, courses and engaging with experts.

The focus of both the FEBS AWS by examination and the honorary certificate is centred around the countries served by the UEMS. However, surgeons from non-European countries can obtain the title FEBS AWS, if they can provide proof of meeting the eligibility requirements and pass the examination, or be considered meritous of an honorary certificate.

In summary, the UEMS Section of Surgery together with the European Board of Surgery and the EHS have established the first and only structured training and examination concept for qualification in AWS. AWS is now firmly established as a subspecialty of General Surgery.

## Footnotes

1
https://uemssurg.quiz.one/
